# Meteorologically estimated exposure but not distance predicts asthma symptoms in schoolchildren in the environs of a petrochemical refinery: a cross-sectional study

**DOI:** 10.1186/1476-069X-8-45

**Published:** 2009-09-25

**Authors:** Neil White, Jim teWaterNaude, Anita van der Walt, Grant Ravenscroft, Wesley Roberts, Rodney Ehrlich

**Affiliations:** 1 School of Public Health and Family Medicine, University of Cape Town, Observatory 7925, Cape Town, South Africa; 2 Asbestos Relief Trust, PO Box 34560, Groote Schuur 7937, Cape Town, South Africa; 3 Ecoserv, 112 Buitengracht St, Cape Town 8000, South Africa; 4 Council for Scientific and Industrial Research, PO Box 320, Stellenbosch 7599, South Africa

## Abstract

**Background:**

Community concern about asthma prompted an epidemiological study of children living near a petrochemical refinery in Cape Town, South Africa. Because of resource constraints and the complexity of refinery emissions, neither direct environmental measurements nor modelling of airborne pollutants was possible. Instead a meteorologically derived exposure metric was calculated with the refinery as the putative point source. The study aimed to determine whether (1) asthma symptom prevalences were elevated compared to comparable areas in Cape Town and (2) whether there was an association between asthma symptom prevalences and the derived exposure metric.

**Methods:**

A cross-sectional study was carried out of all consenting school children aged 11 to 14 years attending schools in a defined area, utilizing the International Study of Asthma and Allergy in Childhood (ISAAC) written and video questionnaires. Information was collected on potential confounders, e.g. parental history of atopic disease, active and passive smoking by the participant, birth order, number of children in the home and distance from a major road. The exposure metric combined residential distance of each child from the refinery with a wind vector in the form of wind speed, wind direction and proportion of the year blown.

**Results:**

A total of 2,361 children from 17 schools met the criteria for inclusion. In multivariate analysis, meteorologically estimated exposure (MEE), but not simple distance from the refinery, was positively associated with having to take an inhaler to school [odds ratio per interquartile range (OR) 1.22, 95% confidence interval (CI) 1.06-1.40], and with a number of video elicited asthma symptoms, including recent waking with wheezing (OR 1.33, 95% CI 1.06-1.66) and frequent wheezing at rest (OR 1.27, 95% CI 1.05 - 1.54). Symptom prevalences were higher than in other areas of the city, with frequent waking with wheezing being in great excess (OR 8.92, 95% CI 4.79-16.63).

**Conclusion:**

The results support the hypothesis of an increased prevalence of asthma symptoms among children in the area as a result of refinery emissions and provide a substantive basis for community concern. The methodology also provides a low cost means of testing hypotheses about point source pollutant effects on surrounding populations of children.

## Background

The investigation of the health impact of air pollution from point sources in resource poor settings poses a number of difficulties, the most formidable of which is measurement of exposure. Longstanding and unresolved community complaints of ill health, and particularly of childhood asthma, linked to emissions from a large petrochemical refinery in Cape Town, South Africa, offered an opportunity to conduct a study using modern but relatively low cost epidemiologic techniques.

The refinery's impact on the health of the community has been the subject of contention. An early study of the area conducted in 1984 found no increased rates of visits to primary care practitioners for upper respiratory tract ailments compared to control areas [1]. By contrast, a later study demonstrated a higher prevalence of bronchial hyperresponsiveness to histamine among schoolchildren in the area than in a control area [2].

A number of studies have demonstrated an association between respiratory symptoms, asthma diagnosis or exacerbation and lung function deficit in children, and residential proximity to industrial processes including power and petrochemical plants [3-7]. Petrochemical refinery emissions are complex and heterogeneous, consisting of particulates, gases or particulates with gases absorbed onto them. Refineries also vary in their emissions depending on many factors [8-10]. Emissions include sulphur dioxide (SO_2_), particulates and oxides of nitrogen as well as fugitive emissions consisting of numerous aliphatic and aromatic hydrocarbons [8]. SO_2 _is of particular concern [7,10].

The study described here had two objectives. The first was to determine whether asthma symptom frequency among children in the area was associated with an exposure metric reflecting refinery emissions. Symptom frequency was derived from the International Study of Asthma and Allergies in Childhood (ISAAC) questionnaires [11]. The limited budget for the study, together with the complexity of deciding which emissions to measure, required a method not reliant on physical or environmental monitoring or modelling to assign exposure [12]. Instead, mathematical modelling in the form of a meteorologically estimated exposure index linked to each participant's residential address was used, with the refinery as the putative point source. Construction of such an index was made easier by the fact that the study area has well characterised meteorological conditions recorded in the vicinity of the refinery.

The second objective of the study was to determine whether children in the area had a higher prevalence of asthma symptoms than children in other parts of the city. Cape Town was a collaborating centre in Phase III of the ISAAC conducted in the same year as but independently of the study reported here [13,14]. This provided a means of comparing asthma symptom prevalences in the study area with those of other areas in the city of similar socioeconomic status.

## Methods

### Study subjects and design

The intention of the study was to enroll all children of appropriate age who both lived and attended school in the quarter of the city of interest (defined as north of Boundary Road in the Cape Town suburb of Milnerton and the N1 motorway leading out of the city). The area is mainly of higher socioeconomic status, with the exception of two areas of low cost or informal housing.

No further sampling was done. In order that more than 3,000 participants from this restricted area could be enrolled (to meet the ISAAC sample size requirement for comparisons of symptom severity [11]), the age range for enrolment was widened to 11 to 14 years from the 13 to 14 years in the ISAAC protocol.

### Outcome and covariate exposure measurement

This study used the ISAAC video and written questionnaires with additional questions on number of siblings, relative birth order of the participant, passive smoking (smoking by any adult in the home), having ever tried active smoking, family members with asthma or hayfever (family atopy) and need to take an asthma inhaler to school. Questionnaires were available in the three local languages, English, Afrikaans and Xhosa. The study was conducted between February and August 2002.

### Exposure in relation to refinery

The exposure metric was constructed to include wind direction and speed, as well as Geographic Information System (GIS) plotted residential straight line distance from the putative point source.

**Figure 1:**
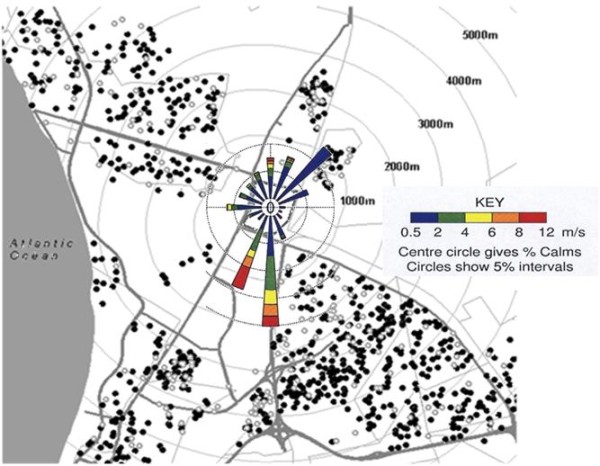
**Distribution of responses to video question "frequent wheeze" in relation to refinery; with wind rose**. The figure shows a map of the area around the refinery, oriented with the Atlantic Ocean to the west. Concentric rings represent distance in meters from the perimeter of the refinery, and heavy lines indicate main roads. Overlying the refinery at its centre is the wind rose for 2001. Wind direction is predominantly from the south. For illustrative purposes, video responses based on question 1c (frequent wheeze) are represented by closed circles (positive responses) and open circles (negative responses).

Respondents were asked to provide a precise home address in order to plot this using a GIS street map of Cape Town. Where a GIS match could not be made by computer (n = 86), the address was entered manually from a street directory. GIS plotting was used to estimate three additional variables for each participant: distance in meters from the nearest major road, distance in meters from the midpoint of the refinery and compass bearing from the refinery. As illustrated in Figure 1, the refinery is surrounded by residential neighbourhoods. At the time of the study it was the sole heavy industry in the area.

Meteorological data for the year prior to the study (2001) were obtained from Scientific Services, City of Cape Town, in the form of an annual wind rose from the meteorological station closest to the refinery and sited to the northwest (Figure 1). Cape Town has a Mediterranean climate with predominantly south easterly winds in summer and north westerly winds in winter together with winter rainfall. Inversion conditions are common in Autumn (March to May).

The wind rose provided three variables: wind direction in 16 compass segments, wind velocity (v) at 0.5, 2.0, 4.0, 6.0, 8.0 and 12 m/s respectively, and proportion of the year blown at each given velocity (p). This information was used to estimate an individual's exposure to emissions from the refinery during this period, based on a velocity weighted average of wind exposure on the assumption that exposure was proportional to p and 1/v along each compass segment. Exposure was therefore estimated as the sum of p/v for each compass segment for those residential points downwind of the refinery.

Within each compass segment individual dose was also taken as being inversely proportional to GIS estimated distance in km (d) from the petrochemical refinery. For individual *i *the meteorologically estimated exposure (MEE) to refinery emissions was therefore proportional to the (sum of p/v) × 1/d and calculated as follows:

The unit is s/m^2^, converted in the analysis to hr/km^2^.

### Comparison with Cape Town Phase III ISAAC study

At the time the analysis for this study was carried out, an analysis of a 50 percent random sample (n = 2,226) of the independent ISAAC Phase III study in Cape Town had been completed, with special emphasis on residence based data using a small area "deprivation index" derived from national census data [13]. Participants in that study were classified into one of ten bands based on area deprivation index, following a method developed in the ISAAC Phase I study in Cape Town [15].

In the ISAAC study, video symptom prevalence tended to increase with area deprivation [13]. In order to ensure comparability of socioeconomic status, the schools in the current study were also categorised by small area deprivation index. Fifteen of the seventeen schools were attended mainly by children from lower middle to upper middle class housing areas, and fell into the "upper" six bands on the area deprivation index. The other two schools served children from informal or very low income housing areas. For purposes of comparison, only ISAAC participants who fell into the upper six bands of that study were used as the control group.

### Statistical analysis

Analysis was conducted using Stata 8 (Stata Corp., Texas). GIS coding and map production used Arcview (Vers. 3.1, Environmental Systems Research Institute, California). All geocoded data were subjected to range and manual checks for correctness.

The association between each of the asthma symptoms on the written and video questionnaires and exposure was examined using univariate logistic regression. Distance from the refinery and MEE were entered in separate analyses as continuous exposure variables. The odds ratio is thus interpreted as a change in the odds associated with a one km increment in residential distance from the refinery or a one unit increment in MEE. To allow for the two different units and distributions, odds ratios were also expressed over the interquartile range as the incremental unit. Final models using multivariate regression, for distance and MEE separately, were chosen based on likely and relevant confounders after examination of the findings from univariate analysis.

### Ethics

The governing bodies of all schools in the area were approached and all gave their consent and cooperation for the study, as did the Western Cape Department of Education. Parents of potential participants were sent a letter explaining the nature of the study and giving them the option of withdrawing their child. This study was approved by the Ethics and Research Committee of the Health Sciences Faculty of the University of Cape Town.

## Results

A total of 2,361 children from 17 schools in the area met the criteria for inclusion as participants in the study out of a total of 3,592 who completed questionnaires (overall participation > 96%). Exclusions were for (a) being outside the desired age range 11 to 14 years (189), (b) residence outside the study area (792), (c) having incomplete address information that did not permit geocoding (126) and (d) having substantially incomplete questionnaires (124).

The mean age of participants was 12.6 years (range 11 to 14 years) and 49.3 percent were male. Median residential distance from the refinery was 3.4 km (interquartile range 2.6 to 4.2; range 1.04 to 4.99). Median MEE was 0.19 hr/km^2 ^(interquartile range 0.10 to 0.39).

Results from selected questions in the written questionnaire and video questionnaire are given in Tables 1 and 2. To allow age-specific comparison with ISAAC studies, prevalences for the age group 13 - 14 years are presented separately.

**Table 1:** Written questionnaire responses for selected questions by age group

Written question	Prevalence (all participants) n = 2,361	Prevalence (ages 13 to 14 years) n = 823
Have you had wheezing or whistling in the chest in the last 12 months?	32.8%	32.4%

Have you ever had asthma?	23.7%	24.2%

Do you need to bring an asthma inhaler to school with you?	9.2%	8.9%

In the last 12 months has your chest sounded wheezy during or after exercise?	34.7%	33.6%

In the last 12 months, have you had a problem with sneezing, or a runny or blocked nose when you did not have a cold or the flu?	58.8%	57.3%

Have you ever had hayfever?	63.9%	61.4%

Have any of your brothers or sisters, or your parents ever had asthma or hayfever ("family atopy")?	69.7%	70.9%

Do any of the adults in your home smoke cigarettes ("passive smoking")?	69.7%	64.2%

Have you ever smoked a cigarette?	38.1%	53.8%

**Table 2:** Video questionnaire responses, participants 11 to 14 years (participants 13 to 14 years in parentheses)

Video question	N	(a) Ever	(b) Recent	(c) Frequent
Wheezing at rest (Sequence 1)	2,361 (823)	29.3% (27.8%)	19.1% (17.1%)	9.6% (8.1%)

Waking with wheezing at night (Sequence 2)	2,347 (815)	35.0% (34.2%)	27.2% (27.4%)	14.5% (15.5%)

Wheezing after exercise (Sequence 3)	2,341 (815)	24.8% (23.0%)	14.7% (12.8%)	7.0% (5.1%)

Waking with coughing at night (Sequence 4)	2,345 (817)	30.4% (27.6%)	18.2% (18.2%)	7.8% (7.2%)

Distressing wheezing at rest (Sequence 5)	2,340 (815)	18.3% (14.3%)	11.0% (8.8%)	5.0% (4.0%)

Recent: in the last 12 months. Frequent: at least monthly in the last 12 months.

### Written questionnaire - univariate analysis

A number of the written questionnaire outcomes (self-reported asthma, exercise wheeze and hayfever) showed no relationship with either distance from the refinery nor MEE. Having to take an asthma inhaler to school was strongly positively associated with MEE [odds ratio (OR) 2.65, 95% confidence interval (CI) 1.52-4.62]. Recent wheeze was less prevalent with increasing distance from the refinery (OR 0.89, 95% CI 0.79 - 1.00).

### Video questionnaire - univariate analysis

Each of five ISAAC video sequences requires participants to indicate if they have had the symptom portrayed: (a) ever, (b) in the last 12 months, and (c) at least once a month in the last 12 months. Table 3 provides a key to the questions in each sequence.

**Table 3:** Asthma symptoms, distance from refinery (km) and meteorologically estimated exposure (MEE) (hr/km^2^)* (N = 2,361)

Outcome variable	Exposure variable	Odds ratio	95% CI	IQR standardised odds ratio	IQR standardised 95% CI
**Need to bring inhaler to school**	**MEE**	**2.26**	**1.27 - 4.04**	**1.22**	**1.06 - 1.40**
	Distance	0.93	0.81 - 1.07	0.89	0.72 - 1.11

Ever wheeze at rest (1a)**	**MEE**	**1.81**	**1.18 - 2.79**	**1.15**	**1.04 - 1.28**
	Distance	1.06	0.97 - 1.16	1.10	0.95 - 1.27

Recent wheezing at rest (1b)	MEE	1.62	0.78 - 3.36	1.12	0.94 - 1.34
	Distance	0.93	0.79 - 1.09	0.89	0.70 - 1.14

**Frequent wheezing at rest (1c)**	**MEE**	**2.74**	**1.24 - 6.04**	**1.27**	**1.05 - 1.54**
	Distance	0.95	0.80 - 1.13	0.93	0.71 - 1.21

Ever wake with wheezing at night (2a)	MEE	1.13	0.74 - 1.73	1.03	0.93 - 1.14
	Distance	1.07	0.98 - 1.17	1.11	0.97 - 1.28

**Recent waking with wheezing at night (2b)**	**MEE**	**3.29**	**1.29 - 8.37**	**1.33**	**1.06 - 1.66**
	Distance	0.97	0.82 - 1.15	0.95	0.74 - 1.24

Frequent waking with wheezing at night (2c)	MEE	1.49	0.77 - 2.89	1.10	0.94 - 1.29
	Distance	1.05	0.90 - 1.23	1.08	0.86 - 1.37

Ever wheeze after exercise (3a)	MEE	1.40	0.89 - 2.21	1.08	0.97 - 1.21
	Distance	0.98	0.89 - 1.09	0.98	0.84 - 1.14

Recent wheezing after exercise (3b)	MEE	1.91	0.85 - 4.31	1.17	0.96 - 1.42
	Distance	0.89	0.75 - 1.05	0.83	0.64 - 1.08

Frequent wheezing after exercise (3c)	MEE	1.75	0.73 - 4.20	1.14	0.93 - 1.41
	Distance	0.90	0.74 - 1.10	0.86	0.63 - 1.16

Ever wake with night cough (4a)	MEE	1.20	0.78 - 1.85	1.04	0.94 - 1.16
	Distance	0.96	0.88 - 1.06	0.95	0.82 - 1.09

Recent waking with coughing at night (4b)	MEE	1.74	0.83 - 3.66	1.14	0.96 - 1.36
	Distance	1.00	0.86 - 1.16	1.00	0.79 - 1.26

Frequent waking with coughing at night (4c)	MEE	1.99	0.87 - 4.54	1.18	0.97 - 1.44
	Distance	0.98	0.82 - 1.17	0.97	0.73 - 1.28

Ever distressing wheeze at rest (5a)	MEE	1.36	0.83 - 2.23	1.08	0.96 - 1.21
	Distance	1.01	0.91 - 1.13	1.02	0.86 - 1.20

Recent distressing wheeze at rest (5b)	MEE	1.83	0.75 - 4.49	1.16	0.93 - 1.43
	Distance	0.98	0.81 - 1.18	0.97	0.72 - 1.30

Frequent distressing wheeze at rest (5c)	MEE	2.66	0.94 - 7.55	1.26	0.98 - 1.62
	Distance	0.97	0.77 - 1.21	0.95	0.67 - 1.35

CI: confidence interval. IQR: interquartile range. Recent: in the last 12 months. Frequent: at least monthly in the last 12 months. Associations with confidence intervals which exclude the null in bold.

*Adjusted for passive smoking, family atopy and GIS distance to nearest major road.

** Video sequence number in parentheses.

In univariate analysis, distance from the refinery was not associated with any of the video questionnaire responses. By contrast, MEE was significantly associated with wheezing at rest (sequence 1a), frequent wheezing at rest (1c), recent sleep disturbance by wheezing (2b), frequent sleep disturbance by cough (4c), and frequent distressing wheeze (5c).

Potentially confounding variables which had significant positive associations with a number of the video outcomes included family atopy (1a, 2a, 3a, 4a, 5a) and passive smoking by the participant (1a, 2a, 3a, 4a, 4b, 5a). GIS measured distance from a busy road was inversely associated with fewer of the video questions (1c, 4c, 5c). The association with gender was inconsistent, with some symptoms being reported more frequently by boys and others by girls.

### Multivariate analysis

Multivariate regression was used to analyse the relationship between having to take an inhaler to school, the video questionnaire outcomes and both MEE and distance from the refinery (in separate models), adjusting for the potentially confounding variables of family history of atopic disease, passive smoking and distance from a major road. Adding gender or active smoking to the models did not alter the results. These variables were therefore excluded from the final analysis presented below.

In multivariate analysis there was no consistent relationship with any of the symptom prevalences and distance, in contrast to the consistently positive relationships with MEE (Table 3). The strongest associations with MEE were recent waking with wheezing, frequent wheezing at rest and having to take an inhaler to school. For illustrative purposes Figure 1, based on responses to question 1c (frequent wheezing at rest), shows the distribution of positive and negative responses to this question in relation to major roads and the refinery.

The analysis was repeated after excluding the two low income schools, with no change in the results.

### Comparison with Cape Town ISAAC Phase III study

The cross-city comparison with other areas is presented in Table 4. The ISAAC analysis by area deprivation did not include the first level of each video sequence (i.e. the "ever" question), nor the sequence portraying exercise wheeze; these associations are thus absent from Table 4.

**Table 4:** Video questionnaire responses: current study versus Cape Town ISAAC Phase III*, ages 13 to 14 years

Video Question	N	Recent % (n)	Frequent % (n)
**Wheezing at rest**			

Current study	823	17.1 (141)	8.1 (67)

2002 ISAAC	270	8.9 (24)	4.1 (11)

OR**(95% CI)		2.12 (1.34-3.35)	2.09 (1.09-4.01)

**Waking with wheezing at night**			

Current study	815	27.4 (224)	15.5 (127)

2002 ISAAC	270	4.1 (11)	2.2 (6)

OR (95% CI)		8.92 (4.79-16.63)	8.12 (3.54 - 18.65)

**Waking with coughing at night**			

Current study	817	18.2 (149)	7.2 (59)

2002 ISAAC	270	12.9 (35)	4.8 (13)

OR (95% CI)		1.50 (1.01-2.23)	1.54 (0.83-2.86)

**Distressing wheeze at rest**			

Current study	815	8.8 (72)	4.0 (33)

2002 ISAAC	270	5.9 (16)	1.9 (5)

OR (95% CI)		1.54 (0.88-2.69)	2.24 (0.86 - 5.79)

Recent: in the last 12 months. Frequent: at least monthly in the last 12 months.

* Higher socioeconomic status areas.

**Odds ratio with 95% confidence interval: 2002 study as referent.

As shown in Table 4, the video questionnaire sequences portraying wheeze at rest, night disturbance by wheeze, night disturbance by cough and distressing wheeze elicited consistently higher prevalences of symptoms in this study than in the city wide ISAAC study. Sleep disturbance by wheeze was strikingly more common in the population living in the vicinity of the refinery, with odds ratios over 8.0.

## Discussion

The first finding was that children residing in the areas around the petrochemical refinery reported a higher prevalence of asthma symptoms than children of the same age and socioeconomic status in other areas of the city. No other covariates were controlled for in this part of the analysis and it is possible that some of the excess was due to differences in area characteristics relevant to asthma risk. In particular, the questionnaires recorded high prevalences of both passive smoking and having actively tried smoking. As comparable smoking data were not available from the city wide ISAAC study, exposure to tobacco smoke could be a contributor to the asthma symptom excess in this population.

In addition, there has long been a high level of concern among parents about the effect of emissions from the refinery on the health of their children and it is possible that children in the study area may either over report symptoms or diagnoses or be more aware of lower respiratory symptoms than children in other parts of the city. The ISAAC video questionnaire with its visually prompted symptoms typically produces lower prevalences than the written questionnaire, a feature which may reduce some of the tendency to reporting bias [16].

The influence of area reporting bias would be also reduced if an accurate exposure measure could be applied to the children residing in the area so as to achieve an exposure gradient within the study population. It was not possible to carry out direct ambient pollutant concentration measurement within the study's budget. A larger budget would not have solved the problem of which pollutants to measure given the complex emissions from the refinery. An alternative exposure metric in the form of a mathematical model based on meteorological data was thus used. The concept of an MEE is a thus a mathematical re-statement of the reasonable proposition that an individual's exposure to a putative point source emission is dependant on how far away they live from the source, how often the wind blows towards them from the source, and how hard the wind blows - with stronger winds resulting in greater dilution of emissions.

On theoretical grounds, this metric would be expected to perform better than simple distance from the refinery. This expectation was strongly confirmed in the study, as distance from the refinery alone was not predictive of any symptom, whereas MEE was. A similar principle of "wind adjusted distance", although a different model, was used in a case control study of asthma attacks in children in Puerto Rico living in proximity to a number of industrial plants [5]. In that study, simple proximity to a number of the plants was predictive of asthma attacks, with wind adjustment making a difference only in the case of one plant. Nevertheless, the current study has shown that wind adjustment may make an appreciable difference to inferences about associations of symptoms with point sources.

A number of studies of children living in the vicinity of industrial and petrochemical plants have shown associations between specific pollutants or proximity to the plants and aggravation of asthma or worse respiratory health [3-7], and to a lesser extent with doctor diagnosed asthma [3,6]. Whether asthma can be caused de novo by typically occurring levels of ambient air pollutants is less clear [17]. If such an effect exists, it may involve interactions with airborne sensitisers [18,19]. In this study, while there was no association between MEE and reported asthma or hayfever, strong associations were found with having to take an asthma inhaler to school, frequent waking at night with wheezing and recent wheezing at rest. The findings are thus compatible with the hypothesis that the effect of refinery emissions is to aggravate asthma.

Potential confounding factors that were controlled in the analysis included passive smoking and family atopy (reported family history of asthma or hayfever), both of which were common in this population. Distance from a major road was included as a proxy for traffic exposure.

Other potential environmental exposures which could vary by area and account for higher respiratory symptoms are aeroallergens. Annual monitoring of pollen counts over a period which included the study dates in both this area and a comparison suburb 10 km away showed some differences [20]. A very strong grass pollen peak was recorded in October (spring), i.e. after the study period, in the study area compared to the control area. The counts of certain other pollens derived from trees, weeds and fungal spores were higher in the control area. As pollen data were not available for most of the areas covered by the 2002 ISAAC study, no inference about the role of pollen as a potential confounding factor could be drawn.

It was not possible in this study to identify which component of refinery admissions might be responsible for symptom aggravation, or alternatively whether a mixture was responsible.

Environmental monitoring of indicator pollutants has been carried out at sites near the refinery for a number of years by the local authority [21]. At the instigation of a local air monitoring task group, monthly reports were produced at the time of the study for three sites, one to the northwest of the petrochemical refinery (Table View), one to the southeast (Bothasig) and a third moveable monitor close to the refinery (Killarney). In addition to SO_2_, particles with an aerodynamic diameter < 10 um (PM_10_), oxides of nitrogen and hydrogen sulphide are continuously monitored.

SO_2 _has long been the focus in the ongoing concern about the air pollution impact of refinery operations. The Department of Health of the City of Cape Town has been using United Kingdom standards for SO_2 _to report air quality since 2000 [22], particularly in the form of guideline exceedances. For example, a short-term guideline level for SO_2 _of 266 ug/m^3 ^(100 ppb) as a 15 minute mean was adopted. Mean data were available only as monthly means.

In the year of the study, SO_2 _monthly means in the area averaged below 10 ug/m^3 ^(3.75 ppb), well below the guideline annual mean SO_2 _of 20 ug/m^3 ^(8 ppb) [23]. However, short term exceedances are of greater interest. The refinery in question produced up to eighteen tons per day during the year of the study. The air monitoring data for 2002, the year of the study, show 38 exceedances of the 15 minute guideline at the Killarney monitoring site closest to the refinery [21]. The highest level recorded was 605 ug/m^3 ^(227 ppb). From the experience of year on year monitoring from 2002 onwards, it has been observed that a number of these exceedances occur during operational maintenance at the refinery. Maintenance shut-downs at the petrochemical refinery have also been recorded to result in significant lowering of monthly mean SO_2 _concentrations [23].

It is thus possible that SO_2 _peaks against a background of relatively low average SO_2 _concentrations might underlie the asthma symptom excess and geographic pattern noted in this study. In a recent study of asthma hospitalisations and emergency room visits among children living near two petroleum refineries in Montreal, Smargiassi et al. [7] found same day SO_2 _peaks to be a better predictor of such asthma episodes than daily SO2 means. However, even in studies where SO_2 _effects have been observed, these may be difficult to distinguish from the effects of co-pollutants such as particulates [24,25]. During the study year, mean monthly PM_10 _concentrations in the area averaged around 30 ug/m^3 ^- again below the guideline annual mean of 40 ug/m^3 ^[23]. Particles with an aerodynamic diameter < 2.5 um (PM_2.5_), a component of refinery emissions [7], were not measured. However, an earlier citywide source apportionment study had found PM_2.5 _levels to be no higher in the study area than in other parts of the city [26].

## Conclusion

The study was conducted following a request from community representatives for an epidemiological study because of concern about possible adverse respiratory health effects on children of the petrochemical refinery's emissions in the surrounding neighbourhoods. It was not designed to link symptom prevalences to specific pollutants or pollution incidents. The study showed a measurable health effect, both an excess of asthma symptoms in comparison with the rest of the city, and more frequent wheezing symptoms associated with an index of estimated petrochemical refinery emissions exposure. These findings thus provided a substantive basis for community concern and for ongoing measures to monitor closely and control emissions from the refinery.

This study demonstrates how measurement of the ISAAC wheeze symptoms, given sufficient sample size, is a practical method of investigating possible health effects from localised sources of emissions. It also illustrates that mathematical dose modelling based on local meteorological data can be used to test hypotheses about putative point source emissions as a risk factor for respiratory ill health, controlling for other known risk factors.

## Abbreviations

SO_2_: sulphur dioxide; ISAAC: International Study of Asthma and Allergies in Childhood; MEE: meteorologically estimated exposure; GIS: geographic information system; PM_10_: particles with an aerodynamic diameter < 10 um; PM_2.5_: particles with an aerodynamic diameter < 2.5 um.

## Competing interests

The authors declare that they have no competing interests.

## Authors' contributions

NW designed and directed the study and prepared the study report before his untimely death in 2004. JteWN devised the MEE metric and managed and analysed the data. RE provided epidemiological expertise to the respiratory survey and wrote the final manuscript. AvdW carried out the respiratory field survey. GR provided expertise on all aspects of ambient pollutant measurement and control. WR carried out the GIS processing. All authors reviewed and approved the final manuscript.
